# Burden analysis of uterine fibroids and endometriosis in reproductive-age women globally, in China, India, and the United States, 1990–2021

**DOI:** 10.3389/fnut.2025.1683847

**Published:** 2026-01-13

**Authors:** Yaru Chen, Qianyan Li, Mengjun Liu, Ying Niu, Hongli Liu, Lu Zhang

**Affiliations:** 1 Department of Gynecologic Oncology, The First Affiliated Hospital of Bengbu Medical University, Bengbu, Anhui, China; 2 Department of Basic Medical Sciences, Qinghai University Medical College, Xining, Qinghai, China

**Keywords:** endometriosis, global disease burden, health inequality, SDI, uterine fibroids, women of childbearing age

## Abstract

**Background:**

Uterine fibroids and endometriosis are common benign gynecological diseases affecting the health of women of childbearing age, characterized by high incidence and recurrence rates. Despite the increasing global emphasis on women’s health, there is still a lack of systematic research on the burden these diseases impose on healthcare systems and society. This study is the first to comprehensively assess the disease burden on women of childbearing age globally, as well as in China, India, and the United States, from 1990 to 2021, and to predict future trends.

**Methods:**

Based on the Global Burden of Disease Study 2021 (GBD 2021) data, the incidence and age-standardized incidence rate (ASIR), disability-adjusted life years (DALYs), and age-standardized DALY rate (ASDR) were analyzed. Methods such as joinpoint regression, decomposition analysis, health inequality indices, and Bayesian age-period-cohort (BAPC) models were used to predict disease trends up to 2036.

**Results:**

In 2021, the global ASIR for uterine fibroids was 250.93 per 100,000 [showing an upward trend, estimated annual percentage change (EAPC) = 0.24], while the ASIR for endometriosis was 88.52 per 100,000 (showing a downward trend, EAPC = −1.00). The number of cases in China, India, and the United States accounted for nearly half of the global total. Decomposition analysis shows that population growth is the main cause of the burden of uterine fibroids, while epidemiological changes have alleviated the burden of endometriosis. The health inequality analysis indicates that the disease burden is heavier in high-Socio-Demographic Index (SDI) populations. Predictions show that by 2036, the global burden of uterine fibroids will continue to grow, while the burden of endometriosis will generally decline (except in the United States).

**Conclusion:**

The burden of uterine fibroids continues to increase, and although the burden of endometriosis has decreased, it remains severe. Targeted interventions are needed, including early screening, equitable distribution of medical resources, and country-specific strategies, to eliminate health inequalities and mitigate the long-term impact of these diseases on global women’s health.

## Introduction

Gynecological diseases have a significant impact on women’s health. Although global attention is focused on women’s health, a report by the World Health Organization (WHO) in 2022 indicates that progress towards achieving women’s health goals remains slow (1). Uterine fibroids and endometriosis are common benign gynecological diseases that affect women’s health (2, 3). Uterine fibroids, also known as uterine smooth muscle tumors or fibroids, are the most common benign uterine tumors in women. They are the main cause of the disease in reproductive-aged women, with an incidence rate as high as 68.6% (4, 5). They can cause many side effects to women’s lives, including irregular menstruation and abnormal uterine bleeding, severe anemia, dysmenorrhea, and even infertility, premature birth, and recurrent miscarriage (6). Endometriosis is also a common, chronic, and non-fatal gynecological disorder that affects approximately 5–10% of women of childbearing age worldwide. Its clinical symptoms include infertility, dysmenorrhea, and non-menstrual pelvic pain, especially affecting the mental and psychological health of patients (7, 8).

Uterine fibroids and endometriosis have the characteristics of high prevalence and high recurrence rate, which have long-term adverse effects on women’s health (9). Clinically, uterine fibroids treatment is diverse but challenging, with options including surgery (myomectomy and uterine artery embolization), and medications (gonadotropin-releasing hormone agonists). Endometriosis treatment involves estrogen-suppressing hormonal therapy, lesion-removing laparoscopic surgery, and assisted reproductive technology for infertility, yet high recurrence rates and long-term complications remain key clinical hurdles. With the growth of the global female population and the development of society, these diseases not only severely affect the quality of life of patients but also impose a considerable burden on the health care systems and societies of various countries (2, 10).

Compared with previous studies focusing solely on benign gynecological diseases (11, 12), we utilized the Global Burden of Disease Study 2021 (GBD 2021) database to integrate new data sources and improved methods to provide the latest estimates through a combined analysis of multiple diseases. And epidemiological data from global sources, as well as from China, India, and the United States, were selected. These countries not only represent the major populations of developing and developed countries but also reflect different levels of Socio-Demographic Index (SDI). Our study of disease correlations benefits from these differences, as they offer valuable insights for developing precise and localized epidemiological strategies for benign gynecological diseases. Our goal is to assess the incidence and disability-adjusted life year (DALY) trends and burdens of these diseases at the global and national levels through various analytical methods such as SDI, age, year, joinpoint, decomposition analysis, health inequality, and the Bayesian age-period-cohort (BAPC) model future projections, to better understand and address the challenges of global gynecological diseases.

## Methods

### Data sources

The GBD 2021 database on the global burden of diseases, injuries, and risk factors is a valuable resource. It comprehensively presents the health-related obstacles faced by people around the world in the 21st century. It provides extensive data on 371 diseases and related risk factors in 204 countries and regions, which can be accessed through the IHME website.^1^ We obtained the annual age-standardized incidence rate (ASIR), age-standardized DALY rate (ASDR), demographic information, and corresponding SDI indicators related to uterine fibroids and endometriosis in reproductive-age women at the global and national levels. SDI is a comprehensive indicator used to summarize the social and demographic development of a region. The score of SDI ranges from 0 to 1 and is a comprehensive geographical development indicator based on national per capita income, total fertility rate, and average education level (13). In this study, the diagnosis of uterine fibroids is based on the International Classification of Diseases, Tenth Edition (ICD-10), and is coded as D25. The diagnosis of endometriosis is based on the International Classification of Diseases, Tenth Edition (ICD-10), and is coded as (N80–N80.9). The 95% uncertainty intervals (UIs) in the tables and figures are derived from the GBD 2021 database.

### Data analysis

This study employed descriptive analysis to compare the incidence numbers, DALYs, ASIR, ASDR, and the changing trends of EAPC of uterine fibroids and endometriosis globally, in China, India, and the United States in 1990 and 2021. The related trends of uterine fibroids and endometriosis in different age groups during the reproductive years were analyzed. All data used in this study were statistically analyzed using R software (version 4.4.2). Age-standardized rates (ASRs) take into account the influence of age structure, enabling fair comparisons of disease burdens across different regions or time periods, and thereby more accurately reflecting trends in population health (14). *Y* = *α* + *βX* + *ε*, where *Y* represents ln (ASR), *X* represents the calendar year, and *β* determines the positive or negative trend of ASR. The formula for calculating EAPC is EAPC = 100 × [exp(*β*) − 1], and its 95% confidence intervals (CIs) are also derived from the linear model (15).

### Joinpoint analysis

The burden trends of uterine fibroids and endometriosis were analyzed using joinpoint regression. Trends were represented by connecting several different line segments at the joinpoint, and the points of the linear slope with statistically significant changes over time were identified (16). During the period from 1990 to 2021, it is expressed by the average annual percent change (AAPC). The slope of each line segment was expressed in terms of annual percentage change (APC), and the best-fitting model was adopted (17). In this study, we used APC to describe the ASIR, ASDR of uterine fibroids and endometriosis from 1990 to 2021. We used the Joinpoint Regression Software (Version 4.1.0), developed by the National Cancer Institute of the United States.

### Decomposition analysis

Decomposition analysis, as a statistical method, focuses on clarifying the relative roles that different driving factors play in the temporal or spatial variations of relevant disease burden indicators. In this study, the Das Gupta method was employed to break down the changes in ASIR and ASDR of uterine fibroids and endometriosis from 1990 to 2021 into the specific contributions of three factors: aging, population growth, and epidemiological changes. This method can more clearly reveal the influence mechanism for each factor on the trend. Compared with traditional methods such as linear regression, decomposition analysis can isolate the independent effect of each factor on the overall burden change, thereby helping to identify the key drivers of global trends (18).

### Measurement of health inequalities

Extract the ASIR, ASDR for the analysis of health inequalities. In line with the suggestions put forward by the World Health Organization, two typical metrics, specifically the slope index of inequality (SII) and the concentration index (CI), were employed to evaluate both the absolute and relative income-related disparities among countries (19). The SII represents the slope of the regression line that correlates the country-level ASIR and ASDR associated with uterine fibroids and endometriosis to the weighted ranking of each country (20). The CI value of 0 indicates that health outcomes are evenly distributed among different SDI stratified populations; a negative value indicates that they are more concentrated in low-SDI populations, while a positive value indicates that they are more concentrated in high-SDI populations.

### BAPC model

The BAPC model represents an improvement over the traditional age-period-cohort model and is widely used for disease burden prediction. Based on the generalized linear model framework, it estimates the effects of age, period, and cohort on uterine fibroids and endometriosis outcomes. Subsequently, verification and reasoning were carried out using Bayes’ theorem. Based on this, we integrated future population prediction parameters to construct a prediction model to estimate the burden of uterine fibroids and endometriosis (21). In this study, we derived all existing data on uterine fibroids and endometriosis, along with future population prediction data, from the GBD 2021 database.

## Results

### The burden of uterine fibroids and endometriosis globally and in China, India, and the United States

**Figure 1:**
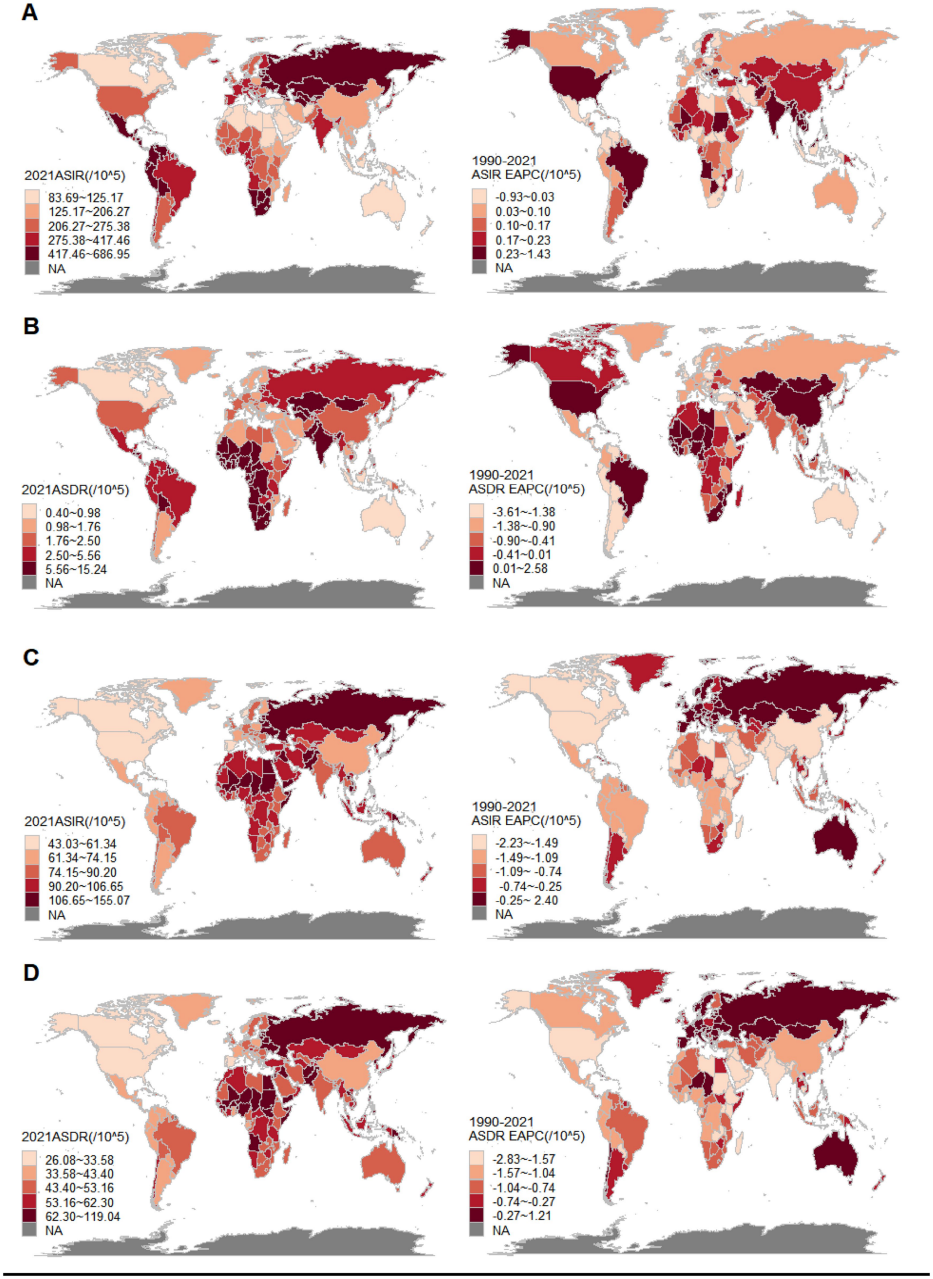
The ASR and EAPC of uterine fibroids and endometriosis for 204 countries. **(A)** The ASIR of uterine fibroids, 2021. The EAPC in the ASIR of uterine fibroids, 1990–2021. **(B)** The ASDR of uterine fibroids, 2021. The EAPC in the ASDR of uterine fibroids, 1990–2021. **(C)** The ASIR of endometriosis, 2021. The EAPC in the ASIR of endometriosis, 1990–2021. **(D)** The ASDR of endometriosis, 2021. The EAPC in the ASDR of endometriosis, 1990–2021.

From a global perspective, the incidence number of uterine fibroids was 10100.27 (95% UI: 7350.44, 13285.68) × 10^3^, and the ASIR was 250.93 (95% UI: 183.44, 330.94) per 100,000 population in 2021. The number of DALYs for uterine fibroids was 142.88 (95% UI: 102.18, 192.99) × 10^3^, and the ASDR was 3.39 (95% UI: 2.43, 4.59) per 100,000 population in 2021. From 1990 to 2021, both the ASIR and ASDR showed an upward trend, with an EAPC of 0.24 (95% CI: 0.23, 0.25) and 0.05 (95% CI: −0.01, 0.11), respectively. In 2021, the incidence number of uterine fibroids in China was 986.13 (95% UI: 712.46, 1285.29) × 10^3^, and the ASIR was 137.25 (101.86, 177.94) per 100,000 population. The number of DALYs for uterine fibroids was 18.59 (95% UI: 9.47, 25.67) × 10^3^, and the ASDR was 1.92 (95% UI: 1.02, 2.67) per 100,000 population. From 1990 to 2021, both the ASIR and ASDR showed an upward trend, with an EAPC of 0.19 (95% CI: 0.06, 0.33) and 2.38 (95% CI: 1.8, 2.98), respectively. In 2021, the incidence number of uterine fibroids in India was 2459.99 (95% UI: 1772.40, 3304.57) × 10^3^, and the ASIR was 326.08 (95% CI: 234.75, 435.25). The number of DALYs for uterine fibroids was 39.68 (95% UI: 27.38, 55.07) × 10^3^, and the ASDR was 5.59 (95% UI: 3.87, 7.73) per 100,000 population in 2021. From 1990 to 2021, ASIR showed an upward trend, with an EAPC of 1.01 (95% CI: 0.88, 1.14), while ASDR showed a downward trend, with an EAPC of −0.42 (95% CI: −0.53, −0.31). The incidence number of uterine fibroids in the United States in 2021 was 428.03 (95% UI: 302.99, 564.89), and the ASIR was 266.67 (95% UI: 190.24, 354.51) per 100,000 population. The number of DALYs for uterine fibroids was 3.58 (95% UI: 2.44, 5.42) × 10^3^, and the ASDR was 1.92 (95% UI: 1.27, 2.88) per 100,000 population. From 1990 to 2021, both the ASIR and ASDR showed an upward trend, with an EAPC of 0.96 (95% CI: 0.56, 1.36) and 0.46 (95% CI: 0.3, 0.62), respectively (Table 1 and Figures 1A,B). From a global perspective, the incidence number of endometriosis was 3447.13 (2436.26, 4611.50) × 10^3^, and the ASIR was 88.52 (62.53, 119.55) per 100,000 population in 2021. The number of DALYs for endometriosis was 2049.47 (1195.20, 3133.97) × 10^3^, and the ASDR was 51.27 (29.87, 78.43) per 100,000 population in 2021. From 1990 to 2021, both the ASIR and ASDR showed a downward trend, with an EAPC of −1.00 (−1.05, −0.95) and −1.01 (−1.06, −0.96), respectively. The incidence number of endometriosis in China was 415.09 (297.59, 554.91) × 10^3^, and the ASIR was 64.95 (46.3, 86.79) per 100,000 population in 2021. The number of DALYs for endometriosis was 269.43 (157.66, 426.16) × 10^3^, and the ASDR was 36.85 (22.02, 58.22) per 100,000 population in 2021. From 1990 to 2021, both the ASIR and ASDR showed a downward trend, with an EAPC of −1.55 (−1.73, −1.36) and −1.53 (−1.72, −1.35), respectively. The incidence number of endometriosis in India was 683.30 (471.06, 924.45) × 10^3^, and the ASIR was 326.08 (234.75, 435.25) per 100,000 population in 2021. The number of DALYs for endometriosis was 392.97 (227.62, 603.47) × 10^3^, and the ASDR was 51.58 (30.06, 78.83) per 100,000 population in 2021. From 1990 to 2021, both the ASIR and ASDR showed a downward trend, with an EAPC of −1.57 (−1.60, −1.53) and −1.73 (−1.77, −1.69), respectively. The incidence number of endometriosis in the United States was 81.02 (58.32, 105.53) × 10^3^, and the ASIR was 53.35 (38.07, 70.03) per 100,000 population in 2021. The number of DALYs for endometriosis was 48.13 (29.69, 73.50) × 10^3^, and the ASDR was 29.92 (18.44, 45.58) per 100,000 population in 2021. From 1990 to 2021, both the ASIR and ASDR showed a downward trend, with an EAPC of −2.13 (−2.37, −1.9) and −1.98 (−2.21, −1.76), respectively (Table 2 and Figures 1C,D).

**Table 1:** The incidence and DALY of uterine fibroids in 1990 and 2021, and their EAPC from 1990–2021.

Region	1990		2021		1990–2021
	cases (×10^3^, 95% UI)	ASR per 10^5^ (95% UI)	cases (×10^3^, 95% UI)	ASR per 10^5^ (95% UI)	EAPC (95% CI)
Incidence					
Global	6009.55 (4390.47, 8011.36)	234.36 (171.06, 309.92)	10100.27 (7350.44, 13285.68)	250.93 (183.44, 330.94)	0.24 (0.23, 0.25)
China	781.28 (557.98, 1068.44)	126.14 (91.36, 171.74)	986.13 (712.46, 1285.29)	137.25 (101.86, 177.94)	0.19 (0.06, 0.33)
India	977.88 (712.19, 1319.81)	256.83 (185.63, 344.16)	2459.99 (1772.40, 3304.57)	326.08 (234.75, 435.25)	1.01 (0.88, 1.14)
United States	306.84 (227.61, 402.10)	212.20 (158.5, 275.35)	428.03 (302.99, 564.89)	266.67 (190.24, 354.51)	0.96 (0.56, 1.36)
DALY					
Global	81.14 (57.13, 111.99)	3.48 (2.46, 4.77)	142.88 (102.18, 192.99)	3.39 (2.43, 4.59)	0.05 (−0.01, 0.11)
China	6.88 (4.15, 12.62)	1.32 (0.8, 2.51)	18.59 (9.47, 25.67)	1.92 (1.02, 2.67)	2.38 (1.80, 2.98)
India	21.33 (12.28, 31.00)	6.47 (3.75, 9.36)	39.68 (27.38, 55.07)	5.59 (3.87, 7.73)	−0.42 (−0.53, −0.31)
United States	2.32 (1.62, 3.52)	1.62 (1.13, 2.45)	3.58 (2.44, 5.42)	1.87 (1.27, 2.88)	0.46 (0.30, 0.62)

**Table 2:** The incidence and DALY of endometriosis in 1990 and 2021, and their EAPC from 1990–2021.

Region	1990		2021		1990–2021
	cases (×10^3^, 95% UI)	ASR per 10^5^ (95% UI)	cases (×10^3^, 95% UI)	ASR per 10^5^ (95% UI)	EAPC (95% CI)
Incidence					
Global	3330.20 (2308.56, 4506.99)	119.65 (83.51, 160.46)	3447.13 (2436.26, 4611.50)	88.52 (62.53, 119.55)	−1.00 (−1.05, −0.95)
China	666.68 (449.76, 920.90)	100.64 (69.31, 139.6)	415.09 (297.59, 554.91)	64.95 (46.3, 86.79)	−1.55 (−1.73, −1.36)
India	596.96 (413.80, 809.53)	141.00 (98.47, 190.17)	683.30 (471.06, 924.45)	88.14 (61.21, 118.86)	−1.57 (−1.60, −1.53)
United States	122.64 (81.15, 173.13)	88.42 (59.4, 125.41)	81.02 (58.32, 105.53)	53.35 (38.07, 70.03)	−2.13 (−2.37, −1.90)
DALY					
Global	1829.34 (1033.45, 2864.71)	69.60 (39.69, 109.37)	2049.47 (1195.20, 3133.97)	51.27 (29.87, 78.43)	−1.01 (−1.06, −0.96)
China	348.78 (193.76, 569.25)	56.72 (32.04, 93.83)	269.43 (157.66, 426.16)	36.85 (22.02, 58.22)	−1.53 (−1.72, −1.35)
India	337.54 (193.23, 518.06)	86.10 (49.5, 133.59)	392.97 (227.62, 603.47)	51.58 (30.06, 78.83)	−1.73 (−1.77, −1.69)
United States	67.17 (37.61, 112.60)	47.48 (26.77, 79.25)	48.13 (29.69, 73.50)	29.92 (18.44, 45.58)	−1.98 (−2.21, −1.76)

### The burden of uterine fibroids and endometriosis in different age groups

**Figure 2:**
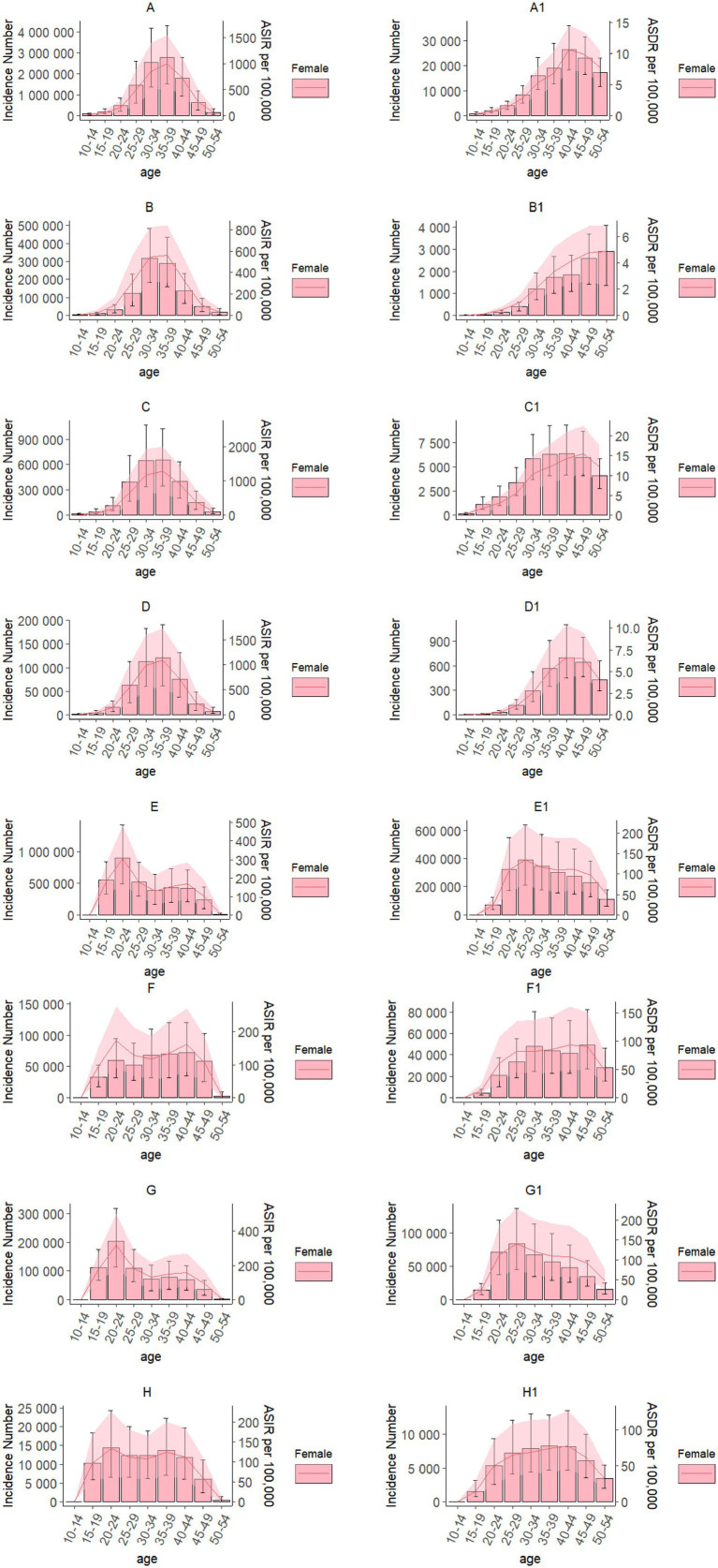
Case numbers and ASR of uterine fibroids and endometriosis among women of reproductive age in 2021. The shaded areas in the figure represent the 95% UI. **(A,A1–D,D1)** Incidence, ASIR, DALYs, and ASDR of uterine fibroids globally and in China, India, and the U.S. **(E,E1–H,H1)** Incidence, ASIR, DALYs, and ASDR of endometriosis globally and in China, India, and the U.S.

Based on the GBD 2021 database, we identified the minimum and maximum ages for the incidence and DALY rates of uterine fibroids and endometriosis as 10 years and 54 years respectively, and divided these ages into 9 age groups with a 5-year interval. In 2021, from a global perspective, among women of childbearing age, both the incidence number of uterine fibroids and the ASIR were highest in the 35–39 age group, at 2791.93 (95% UI: 1512.49, 4312.69) × 10^3^ and 1005.00 (95% UI: 544.45, 1552.43) per 100,000 population, respectively. The number of DALYs and the ASDR were the highest in the 40–44 age group, at 26.47 (95% UI: 18.34, 36.13) × 10^3^ and 10.67 (95% UI: 7.39, 14.56) per 100,000 population, respectively (Figures 2A,A1). The incidence number of uterine fibroids among Chinese women of childbearing age is the highest in the 31–34 age group, at 316.33 (95% UI: 182.10, 482.21) × 10^3^; the ASIR is the highest in the 35–39 age group, at 561.53 (95% UI: 308.19, 841.23) per 100,000 population. The highest number of DALYs and ASDR were both observed in the 50–54 age group, at 2.90 (95% UI: 1.36, 4.10) × 10^3^ and 4.86 (95% UI: 2.28, 6.87) per 100,000 population, respectively (Figures 2B,B1). In India, among women of childbearing age, both the incidence number of uterine fibroids and the ASIR peaked in the 35–39 age group, at 655.29 (95% UI: 344.08, 1028.78) × 10^3^ and 1267.28 (95% UI: 665.43, 1989.57) per 100,000 population, respectively. The number of DALYs was highest in the 40–44 age group, at 6.34 (95% UI: 4.15, 9.33) × 10^3^, while the ASDR was highest in the 45–49 age group, at 15.51 (95% UI: 10.63, 22.55) per 100,000 population (Figures 2C,C1). Among women of childbearing age in the United States, both the incidence number of uterine fibroids and the ASIR peaked in the 35–39 age group, at 121.01 (95% UI: 61.80, 191.40) × 10^3^ and 1089.84 (95% UI: 556.59, 1723.70) per 100,000 population, respectively. The number of DALYs and the ASDR both reached their highest in the 40–44 age group, at 0.70 (95% UI: 0.45, 1.11) × 10^3^ and 6.61 (95% UI: 4.27, 10.42) per 100,000 population, respectively (Figures 2D,D1). In 2021, from a global perspective, among women of childbearing age, both the incidence number of endometriosis and the ASIR were highest in the 20–24 age group, at 893.93 (95% UI: 491.59, 1420.49) × 10^3^ and 304.31 (95% UI: 167.35, 483.57) per 100,000 population, respectively. The number of DALYs and the ASDR were the highest in the 25–29 age group, at 391.77 (95% UI: 212.57, 639.83) × 10^3^ and 134.63 (95% UI: 73.05, 219.88) per 100,000 population, respectively (Figures 2E,E1). The incidence number of endometriosis among Chinese women of childbearing age is the highest in the 40–44 age group, at 71.84 (95% UI: 35.02, 120.77) × 10^3^; the ASIR is the highest in the 20–24 age group, at 173.14 (95% UI: 92.35, 277.42) per 100,000 population. The number of DALYs was highest in the 45–49 age group, at 49.24 (95% UI: 26.72, 82.18) × 10^3^, while the ASDR was highest in the 40–44 age group, at 93.19 (95% UI: 50.33, 161.43) per 100,000 population (Figures 2F,F1). In India, among women of childbearing age, both the incidence number of endometriosis and the ASIR peaked in the 20–24 age group, at 203.49 (95% UI: 115.23, 319.28) × 10^3^ and 319.60 (95% UI: 180.98, 501.45) per 100,000 population, respectively. The number of DALYs and the ASDR were the highest in the 25–29 age group, at 110.83 (95% UI: 62.62, 174.41) × 10^3^ and 185.11 (95% UI: 104.59, 291.28) per 100,000 population, respectively (Figures 2G,G1). Among women of childbearing age in the United States, both the incidence number of endometriosis and the ASIR peaked in the 20–24 age group, at 14.32 (95% UI: 6.44, 24.37) × 10^3^ and 133.60 (95% UI: 60.05, 227.37) per 100,000 population, respectively. The number of DALYs was highest in the 35–39 age group, at 8.27 (95% UI: 4.65, 12.85) × 10^3^, while the ASDR was highest in the 40–44 age group, at 77.64 (95% UI: 44.25, 127.56) per 100,000 population (Figures 2H,H1).

### Joinpoint analysis of uterine fibroids and endometriosis

**Figure 3:**
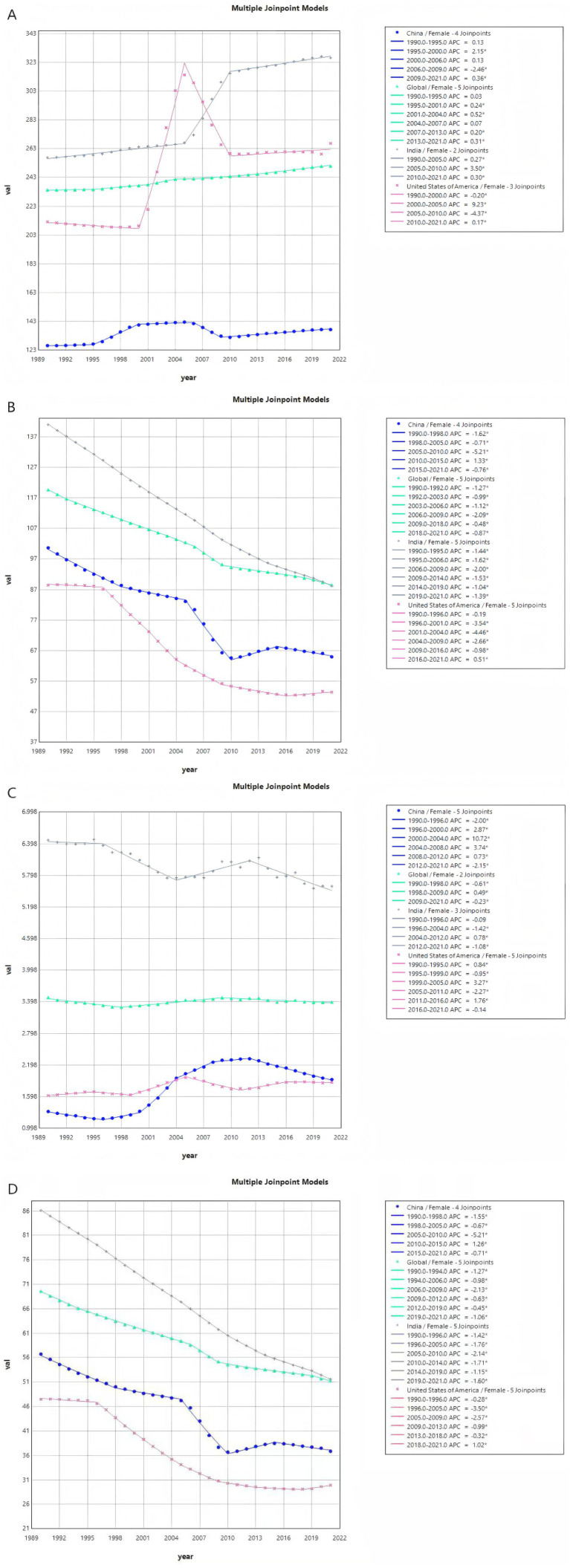
Joinpoint analysis of uterine fibroids and endometriosis. **(A)** ASIR of uterine fibroids from 1990 to 2021. **(B)** ASDR of uterine fibroids from 1990 to 2021. **(C)** ASIR of endometriosis from 1990 to 2021. **(D)** ASDR of endometriosis from 1990 to 2021.

From the perspective of ASIR from 1990 to 2021, the AAPC of global uterine fibroids was 0.23, with *p* < 0.001. In a phased analysis, from 2001 to 2004, the upward trend was relatively rapid, with an APC of 0.52, and *p* < 0.05. The AAPC in China was 0.28, with *p* < 0.001. During the period from 1995 to 2000, the upward trend was relatively rapid, with an APC of 2.15 and *p* < 0.05. The AAPC in India was 0.79, with *p* < 0.01. Among these, the upward trend was relatively rapid from 2005 to 2010, with an APC of 3.5 and *p* < 0.05. The AAPC in the United States was 0.70, with *p* < 0.001. Among these, the increase trend from 2000 to 2005 was relatively rapid, with an APC of 9.23 and *p* < 0.05 (Figure 3A). The AAPC of global endometriosis was −0.97, with *p* < 0.001. In a phased analysis, from 1990 to 1992, the decline trend was relatively rapid, with an APC of −1.27 and *p* < 0.05. The AAPC in China was −1.37, with *p* < 0.001. During the period from 2005 to 2010, the decline trend was relatively rapid, with an APC of −5.21 and *p* < 0.05. The AAPC in India was −1.50, with *p* < 0.01. Among these, the decline trend was relatively rapid from 2006 to 2009, with an APC of −2.00 and *p* < 0.05. The AAPC in the United States was −1.62, with *p* < 0.001. Among these, the decline trend from 2001 to 2004 was relatively rapid, with an APC of −4.46 and *p* < 0.05 (Figure 3B). From the perspective of ASDR from 1990 to 2021, the AAPC of global uterine fibroids was −0.08, with *p* < 0.01. During the period from 1990 to 1998, the decline trend was relatively rapid, with an APC of −0.61 and *p* < 0.05. The AAPC in China was 1.23, with *p* < 0.001. During the period from 2000 to 2004, the upward trend was relatively rapid, with an APC of 10.72 and *p* < 0.05. The AAPC in India was −0.74, with *p* < 0.001. During the period from 1996 to 2004, the decline trend was relatively rapid, with an APC of −1.42 and *p* < 0.05. The AAPC in the United States was 0.31, with *p* < 0.001. During the period from 1999 to 2005, the upward trend was relatively rapid, with an APC of 3.27 and *p* < 0.05 (Figure 3C). The AAPC of global endometriosis was −0.98, with *p* < 0.001. During the period from 2006 to 2009, the decline trend was relatively rapid, with an APC of −2.13 and *p* < 0.05. The AAPC in China was −1.42, with *p* < 0.001. During the period from 1990 to 1998, the decline trend was relatively rapid, with an APC of −1.55 and *p* < 0.05. The AAPC in India was −1.66, with *p* < 0.001. During the period from 2005 to 2010, the decline trend was relatively rapid, with an APC of −2.14 and *p* < 0.05. The AAPC in the United States was −1.53, with *p* < 0.001. During the period from 1996 to 2005, the decline trend was relatively rapid, with an APC of −3.5 and *p* < 0.05 (Figure 3D).

### Decomposition analysis of uterine fibroids and endometriosis

**Figure 4:**
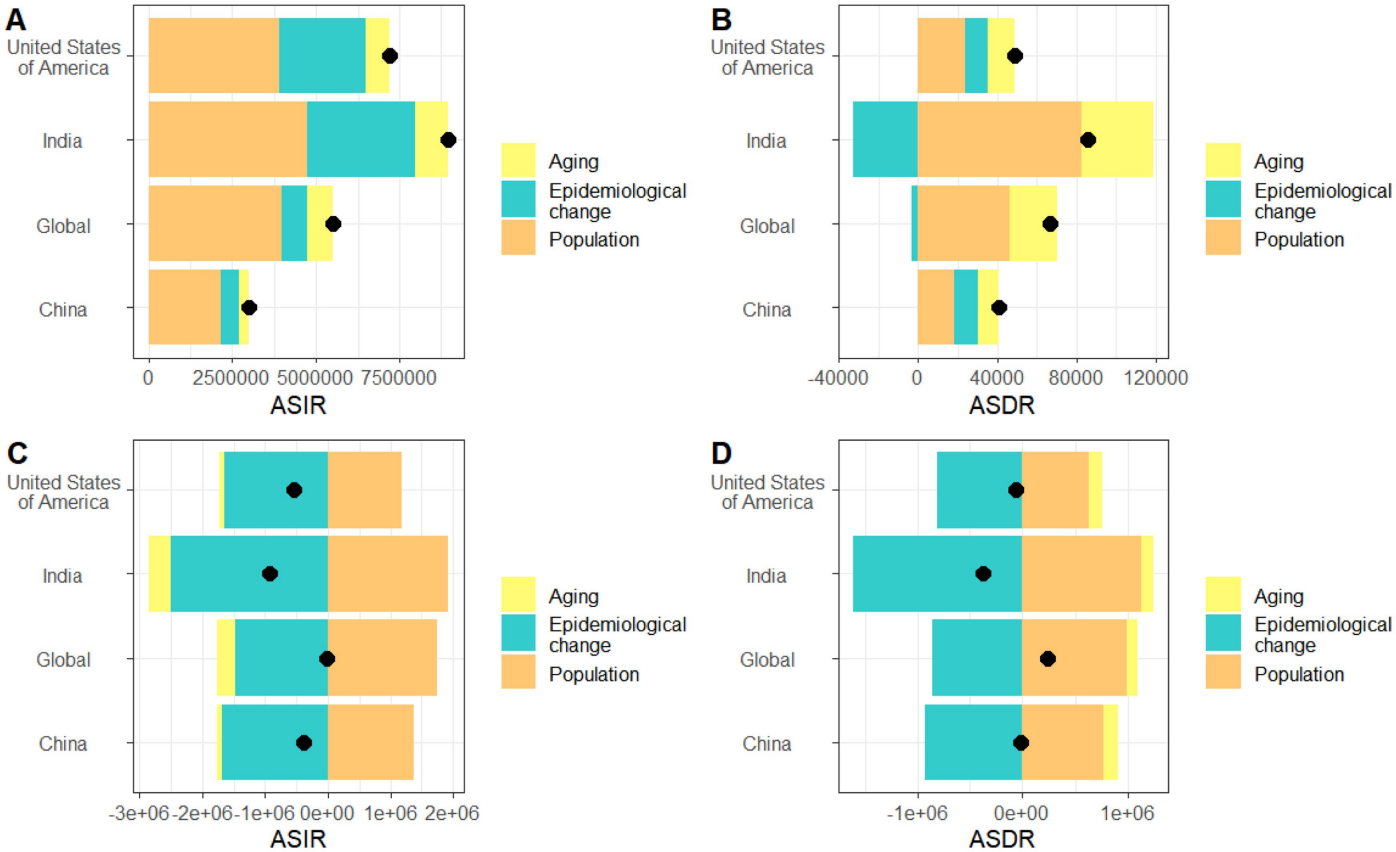
Decomposition analysis results of uterine fibroids and endometriosis-related ASIR and ASDR. **(A,B)** Different decomposition factors for uterine fibroids. **(C,D)** Different decomposition factors for endometriosis. The black dots in the figure represent the total difference effect.

To investigate the effects of population aging, demographic growth, and epidemiological changes on the ASIR and ASDR of uterine fibroids and endometriosis from 1990 to 2021, we conducted a quantitative decomposition analysis. From the perspective of ASIR of uterine fibroids, population growth is a major contributor globally and in China, India, and the United States, increasing cases by 4.00 million (71.84%), 2.16 million (71.77%), 4.76 million (53.06%), and 3.91 million (54.15%), respectively. Followed by epidemiological change and aging (Figure 4A). Similarly, population growth remained the primary contributor to ASDR in these four regions, increasing cases by 46.33 thousand (69.37%), 18.17 thousand (44.66%), 82.38 thousand (95.93%), and 23.87 thousand (48.74%), respectively, followed by aging. It is worth noting that epidemiological changes have played a mitigating role globally and in India (Figure 4B). From the perspective of the ASIR of endometriosis, population growth was the main contributing factor globally and in China, India, and the United States, increasing cases by 1.74 million (−9902.48%), 1.38 million (−355.45%), 1.92 million (−205.73%), and 1.18 million (−218.16%), respectively. The epidemiological changes played a mitigating role, reducing the number of cases by 1.47 million (8375.49%), 1.69 million (435.96%), 2.51 million (268.17%), and 1.65 million (305.88%), respectively (Figure 4C). From the perspective of ASDR of endometriosis, population growth was the main contributing factor globally and in China, India, and the United States, increasing cases by 1.00 million (416.26%), 0.77 million (−4499.47%), 1.14 million (−304.39%), and 0.63 million (−987.07%), respectively. The epidemiological changes played a mitigating role, reducing the number of cases by 0.86 million (−358.42%), 0.93 million (5458.01%), 1.61 million (433.45%), and 0.82 million (1275.79%), respectively (Figure 4D). In the figure, positive values on the abscissa represent promoting factors, while negative values represent mitigating factors. The percentage of each factor is calculated as (the number of effect cases of each factor divided by the total difference effect) × 100%.

### Cross-country analysis of inequalities in uterine fibroids and endometriosis

**Figure 5:**
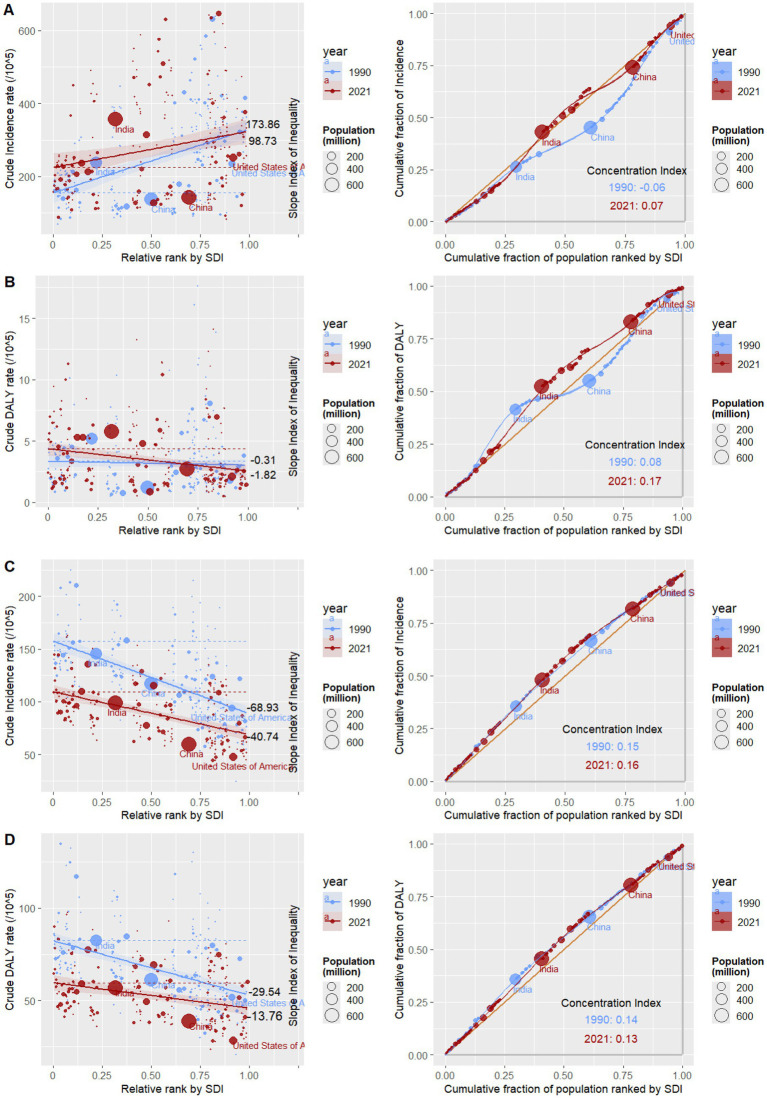
Analysis of health inequalities in uterine fibroids and endometriosis. **(A)** Slope index and concentration index of uterine fibroids crude incidence rates. **(B)** Slope index and concentration index of uterine fibroids crude DALY rates. **(C)** Slope index and concentration index of endometriosis crude incidence rates. **(D)** Slope index and concentration index of endometriosis crude DALY rates.

Absolute and relative inequalities related to SDI were detected in the global burden of uterine fibroids among reproductive-age women. The slope indices of the crude incidence rates were 173.86 in 1990 and 98.73 in 2021 per 100,000 population. This reflects that as the SDI ranking changes, the absolute difference in crude incidence rates is narrowing, meaning that the degree of health inequality in the crude incidence rate of uterine fibroids across the SDI dimension has alleviated. The concentration index of the incidence in 1990 and 2021 were −0.06 and 0.07. This indicates that the incidence burden was more concentrated among the population with a low SDI in 1990, while conversely, it was more concentrated among the population with a high SDI in 2021 (Figure 5A). The slope indices of the crude DALY rates were −0.31 in 1990 and −1.82 in 2021 per 100,000 population. This reflects that as the SDI ranking changes, the absolute difference in the crude DALY rate is widening, meaning that health inequality in the crude DALY rate of uterine fibroids across the SDI dimension has increased. The concentration index of the DALY in 1990 and 2021 were 0.08 and 0.17. This implies that the inequality in DALY burden among populations with different SDI levels has intensified, with high-SDI populations bearing a relatively greater share of the DALY burden (Figure 5B). Absolute and relative inequalities related to SDI were detected in the global burden of endometriosis among reproductive-age women. The slope indices of the crude incidence rates were −68.93 in 1990 and −40.74 in 2021 per 100,000 population. This reflects that as the SDI ranking changes, the absolute difference in crude incidence rates is narrowing, meaning that the degree of health inequality in the crude incidence rate of endometriosis across the SDI dimension has alleviated. The concentration index of the incidence in 1990 and 2021 were 0.15 and 0.16. This implies that the inequality in incidence burden among populations with different SDI levels has intensified, with high-SDI populations bearing a relatively greater share of the incidence burden (Figure 5C). The slope indices of the crude DALY rates were −29.54 in 1990 and −13.76 in 2021 per 100,000 population. This reflects that as the SDI ranking changes, the absolute difference in crude DALY rates is narrowing, meaning that the degree of health inequality in the crude incidence rate of endometriosis across the SDI dimension has alleviated. The concentration index of the DALY in 1990 and 2021 were 0.14 and 0.13. This indicates that DALY burden inequality among SDI groups has decreased, with the burden increasingly concentrated in low-SDI populations (Figure 5D).

### Future projections of uterine fibroids and endometriosis

**Figure 6:**
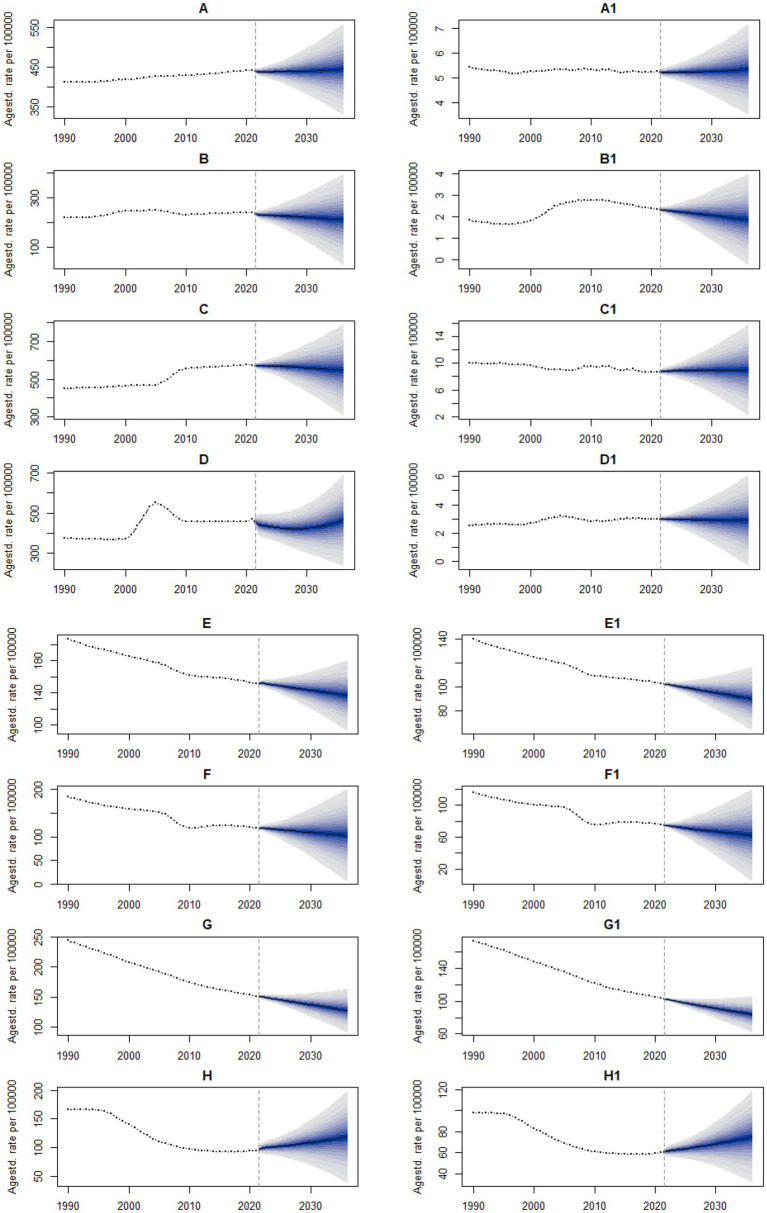
Observed and predicted ASR of uterine fibroids and endometriosis from 1990 to 2036. **(A,A1)** Global ASIR and ASDR of uterine fibroids. **(B,B1)** Chinese ASIR and ASDR of uterine fibroids. **(C,C1)** Indian ASIR and ASDR of uterine fibroids. **(D,D1)** U.S. ASIR and ASDR of uterine fibroids. **(E,E1)** Global ASIR and ASDR of endometriosis. **(F,F1)** Chinese ASIR and ASDR of endometriosis. **(G,G1)** Indian ASIR and ASDR of endometriosis. **(H,H1)** U.S. ASIR and ASDR of endometriosis. The blue region shows the upper and lower limits of the 95% UI.

As shown in the figure, the mean ASIR of uterine fibroids among reproductive-age women globally has been gradually increasing from 1990 to 2021, and it is expected to continue to increase by 2036. By 2036, the mean ASIR is estimated to reach 444.53 per 100,000, representing a 0.71% increase compared to 2021. The mean ASDR of uterine fibroids has been gradually decreasing from 1990 to 2021; however, it is projected to show a slow upward trend by 2036. By 2036, the mean ASDR is estimated to reach 5.35 per 100,000, representing a 1.71% increase compared to 2021 (Figures 6A,A1). The mean ASIR of uterine fibroids among women in China has been gradually increasing from 1990 to 2021; however, it is projected to show a decreasing trend by 2036. By 2036, the mean ASIR is estimated to reach 212.16 per 100,000, representing an 11.92% reduction compared to 2021. The mean ASDR of uterine fibroids has been gradually increasing from 1990 to 2021; however, it is projected to show a decreasing trend by 2036. By 2036, the mean ASDR is estimated to reach 1.87 per 100,000, representing a 20.76% reduction compared to 2021 (Figures 6B,B1). The mean ASIR of uterine fibroids among women in India has been gradually increasing from 1990 to 2021; however, it is projected to show a decreasing trend by 2036. By 2036, the mean ASDR is estimated to reach 549.28 per 100,000, representing a 4.47% reduction compared to 2021. The mean ASDR of uterine fibroids has been gradually decreasing from 1990 to 2021; however, it is projected to show a slow upward trend by 2036. By 2036, the mean ASDR is estimated to reach 8.98 per 100,000, representing a 2.98% increase compared to 2021 (Figures 6C,C1). The mean ASIR of uterine fibroids among women in the United States has been gradually increasing from 1990 to 2021; however, it is projected to show a decreasing trend by 2036. By 2036, the mean ASDR is estimated to reach 463.43 per 100,000, representing a 1.28% reduction compared to 2021. The mean ASDR of uterine fibroids has been gradually increasing from 1990 to 2021; however, it is projected to show a decreasing trend by 2036. By 2036, the mean ASDR is estimated to reach 2.90 per 100,000, representing a 3.33% reduction compared to 2021 (Figures 6D,D1). The mean ASIR of endometriosis among women globally has been gradually decreasing from 1990 to 2021, and it is expected to continue to decline by 2036. By 2036, the mean ASDR is estimated to reach 137.25 per 100,000, representing a 9.69% reduction compared to 2021. The mean ASDR of endometriosis has been gradually decreasing from 1990 to 2021, and it is expected to continue to decline by 2036. By 2036, the mean ASDR is estimated to reach 90.39 per 100,000, representing a 12.04% reduction compared to 2021 (Figures 6E,E1). The mean ASIR of endometriosis among women in China has gradually decreased from 1990 to 2021 and is expected to continue declining by 2036. By 2036, the mean ASIR is estimated to reach 103.31 per 100,000, representing a 13.00% reduction compared to 2021. The mean ASDR of endometriosis has been gradually decreasing from 1990 to 2021, and it is expected to continue to decline by 2036. By 2036, the mean ASDR is estimated to reach 62.51 per 100,000, representing a 17.29% reduction compared to 2021 (Figures 6F,F1). The mean ASIR of endometriosis among women in India has gradually decreased from 1990 to 2021 and is expected to continue declining by 2036. By 2036, the mean ASIR is estimated to reach 128.00 per 100,000, representing a 15.41% reduction compared to 2021. The mean ASDR of endometriosis has been gradually decreasing from 1990 to 2021, and it is expected to continue to decline by 2036. By 2036, the mean ASDR is estimated to reach 84.12 per 100,000, representing an 18.66% reduction compared to 2021 (Figures 6G,G1). The mean ASIR of endometriosis among women in the United States has been gradually decreasing from 1990 to 2021; however, it is projected to show an increasing trend by 2036. By 2036, the mean ASIR is estimated to reach 118.82 per 100,000, representing a 25.34% increase compared to 2021. The mean ASDR of endometriosis has been gradually decreasing from 1990 to 2021; however, it is projected to show an increasing trend by 2036. By 2036, the mean ASDR is estimated to reach 75.08 per 100,000, representing a 24.20% increase compared to 2021 (Figures 6H,H1).

## Discussion

With the growing global recognition of uterine fibroids and endometriosis as significant health threats, their increasing incidence has posed escalating challenges to public health policies and healthcare systems worldwide. In light of this, this study presents the first comprehensive and systematic assessment of the disease burden of uterine fibroids and endometriosis in reproductive-age women at both global and national levels from 1990 to 2021, along with projections of their future trends. In 2021, the global incidence of uterine fibroids in women of childbearing age was 10100.27 thousand cases, with China accounting for 986.13 thousand cases, India for 2459.99 thousand cases, and the United States for 428.03 thousand cases. These three populous countries together accounted for nearly half of the global burden. The global number of DLAY cases is 142.88 thousand cases, with China accounting for 18.59 thousand cases, India for 39.68 thousand cases, and the United States for 3.58 thousand cases. Together, these three countries account for more than half of the global burden. Similarly, in 2021, the global incidence of endometriosis in women of childbearing age was 3447.13 thousand cases, with China accounting for 415.09 thousand cases, India for 683.30 thousand cases, and the United States for 81.2 thousand cases. The global DALY count is 2049.47 thousand cases, with China at 269.43 thousand cases, India at 392.97 thousand cases, and the United States at 48.13 thousand cases. The incidence and DALY counts in these three major countries exceed one-third of the global burden. The impact of uterine fibroids and endometriosis in these populous countries requires extensive attention from global health authorities.

From 1990 to 2021, the global ASIR showed a slow upward trend, while the ASDR showed a slow downward trend. According to the joinpoint analysis, the AAPC values are 0.23 and −0.08, respectively. According to a systematic analysis of the Global Burden of Disease study, changes in age structure, population growth, and age-specific incidence rates may be influencing factors (22). The ASIR and ASDR of endometriosis both show a downward trend. The AAPC was −0.97 and −0.98, respectively. Our results are consistent with previous GBD studies, which also reported a decline in age-standardized rates of endometriosis over time (12), reflecting significant progress in women’s health over the past few decades. In addition, governments around the world have formulated various plans aimed at improving patients’ prognosis through enhanced awareness and education, better clinical management, and research (23, 24). At the national level, there are significant differences in performance among countries. In China, the ASIR and ASDR of uterine fibroids both show an upward trend, with AAPC values of 0.28 and 10.72, respectively. From the age group analysis, it is found that the peak age burden of uterine fibroids in China is later than that in the global, Indian, and the United States. The advancements and widespread adoption of screening and diagnostic technologies, as well as the relatively older age of uterine fibroid patients, may be associated with the increase in ASIR and ASDR. The ASIR and ASDR of endometriosis both show a downward trend, with AAPCs of −1.37 and −1.42, respectively. This is consistent with previous studies (25, 26). In recent years, indicators of health service investment in China, such as the number of health institutions, the number of health personnel, total building area, and fiscal subsidy income, have all seen varying degrees of growth (27). The convenience and fairness of healthcare services enable Chinese residents to access more and better healthcare services (28). The improvement of the health insurance system and the increase in coverage have, to some extent, alleviated their disease burden (29). The ASIR of uterine fibroids in India shows an upward trend, while the ASDR shows a downward trend, with AAPC values of 0.79 and −0.74, respectively. The ASIR and ASDR of endometriosis both show a downward trend, with AAPC values of −1.50 and −1.66, respectively. As a populous country and a rapidly developing economy, India’s increasing trend of uterine fibroids has had a profound impact worldwide. The rising incidence of uterine fibroids year by year may be associated with the continuous improvement of medical resources and equipment, as well as the heightened awareness of individual health, with these factors coinciding with increased diagnostic rates. In the past two decades, with the rapid economic development and progress in the construction of primary healthcare systems, India has made certain advancements in reducing the ASIR and DALY rates of endometriosis among women (30). The ASIR and ASDR of uterine fibroids in the United States both show an upward trend, with AAPCs of 0.70 and 0.31, respectively. The ASIR and ASDR of endometriosis both show a downward trend, with AAPCs of −1.62 and −4.46, respectively. According to reports, the number of Black women in the United States is high, and the incidence and DALY rates of uterine fibroids among Black women are 3–4 times higher than those of other races. 70–80% of Black women will experience uterine fibroids in their lifetimes (4, 31). Moreover, obesity is also an important factor. Studies have shown that for every additional kilogram of overweight, the risk of developing uterine fibroids increases (32, 33). Research has found that uterine fibroids are more common among African Americans. The obesity rate is highest among the African American population in the United States (34). The decline in the disease burden of endometriosis may be related to advanced medical technology in the United States and increased health awareness among the population. Additionally, a large cohort study conducted by Viganò et al. (35) (Oxford Family Planning Association Study) showed that the use of oral contraceptive pills (OCP) was found to be a protective factor against endometriosis. This may be due to the progestins in birth control pills, which can inhibit the growth of the endometrium, keeping it in a relatively inactive state. From an age perspective, the peak age for DALYs of uterine fibroids during the reproductive period in the two developing countries (China and India) is relatively later compared to the developed country (the United States). This may be related to the traditional diets in China and India, which are rich in vegetables and grains, whereas the diet structure in Western developed countries like the United States is higher in fat and protein. Research indicates that the increase in the consumption of animal protein, particularly meat, may be another factor contributing to the increased risk of uterine fibroids (4, 36). In addition, fertility rates and healthcare standards are also factors. In the past, under traditional beliefs, China and India had higher birth rates, and the hormonal changes in women during pregnancy and breastfeeding can inhibit tumor growth. In China and the United States, the peak age for the DALYs rate of endometriosis during reproductive age is significantly later than the global and Indian rates. This may be related to the postponement of childbearing and the impact of treatment interventions. Women in China and the United States generally have kids later, while women in India tend to have kids earlier. Delaying childbirth may extend the duration of hormone exposure, thereby shifting the peak period of the disease. In terms of treatment, patients in China and the United States are more likely to receive long-term medication (such as GnRH agonists) or surgical interventions, which delay disease progression (37). However, Indian patients may experience rapid deterioration of the disease in early reproductive age due to economic or medical constraints, leading to delayed treatment and an earlier peak in DALYs (12).

Using decomposition analysis, the main factors affecting uterine fibroids and endometriosis during the reproductive period were studied. This research indicates that globally, as well as in China, India, and the United States, population growth may be a primary factor potentially linked to the burden of uterine fibroids and endometriosis, with this primary association particularly coinciding with increases in ASIR and ASDR. It is noteworthy that epidemiological changes show a mitigating effect on the ASDR of uterine fibroids globally and in India. Similarly, epidemiological changes show a relieving effect on the burden of endometriosis globally and in China, India, and the United States, specifically with reductions in ASIR and ASDR, which is consistent with previous studies (12). This positive development trend indicates that public health actions and medical interventions are making commendable progress. At the same time, given the unique characteristics of different countries, tailored intervention methods should be adopted to alleviate the disease burden caused by uterine fibroids and endometriosis.

The health inequality analysis utilizes the GBD 2021 database, aiming to provide information for policies and programs addressing health inequality issues (including unfair, avoidable, or remediable health disparities) (38, 39). The global crude incidence rate CI of uterine fibroids increased from −0.06 in 1990 to 0.07 in 2021, and the crude DALY rate CI increased from 0.06 in 1990 to 0.17 in 2021. This indicates that health inequality distribution is widening, and the burden on high-SDI populations is greater. The global crude incidence rate CI of endometriosis increased from 0.15 in 1990 to 0.16 in 2021, while the crude DALY rate CI decreased from 0.14 in 1990 to 0.13 in 2021. Although the differences are not significant, they indicate that the disparities in distribution across different socioeconomic backgrounds are still widening. It is worth noting that the burden of uterine fibroids and endometriosis in China and the United States has become more evenly distributed in terms of health inequality from 1990 to 2021, while the distribution of health inequality burden in India has expanded, showing a trend towards concentration in high-SDI populations. This reflects the inequality in medical resources and development. These findings indicate the need to address the health inequalities faced by patients with uterine fibroids and endometriosis, particularly in high-SDI level populations. However, we cannot ignore the low-SDI populations, where a lack of medical resources, a large number of undiagnosed mild cases, and delays in drug and surgical interventions may be indirectly associated with the incidence and DALY rates in high-SDI populations. This is consistent with previous studies (40).

In the next 15 years, the disease burden of uterine fibroids among reproductive-age women globally is expected to rise. The decomposition analysis indicates that the primary driving factor is population growth. However, in China, India, and the United States, the disease burden is generally on a downward trend. This may be related to medical advancements, increased health awareness, and improved lifestyles, in line with economic and social development. In the next 15 years, the disease burden of endometriosis among women of childbearing age will show a declining trend globally, in China, and in India. Combined with the results of the decomposition analysis indicate that epidemiological changes have significantly contributed to alleviating this burden. This indicates that the continuous investment by governments and health departments in research, healthcare system construction, and treatment accessibility is of great significance for reducing the long-term burden of endometriosis and improving the quality of life of affected women (41). It is worth noting that the predicted burden of endometriosis in the United States over the next 15 years shows an upward trend. This may be related to delayed childbirth and fewer births in the United States, which could increase the risk of developing endometriosis, thereby leading to a rise in future disease burden. Additionally, obesity and genetic factors contribute to the increasing burden of endometriosis. However, overall, the disease burden of endometriosis remains high. Since endometriosis is progressive, early diagnosis and treatment, especially screening and intervention for patients in the peak age range of reproductive years, are particularly important (42, 43).

### Limitations

This study has some limitations. Firstly, despite the high reliability and accuracy of the official GBD 2021 data we adopted, there may be data quality differences in countries with a low SDI. Their limited access to healthcare services may impede the full screening and accurate diagnosis of uterine fibroids and endometriosis, and the number of cases may be underreported or omitted. Secondly, the diagnostic criteria for uterine fibroids and endometriosis in the data sources of various countries may vary, which affects the accuracy of the estimations in the GBD database. Thirdly, the GBD database lacks detailed info like pathological types, lesion sites, and drug treatments, thus limiting in-depth analysis of uterine fibroids and endometriosis burden. Finally, although the prediction model we constructed has adequate robustness, the development trend of uterine fibroids and endometriosis in the next decade or so is likely to change due to the iteration of medical infrastructure, the adjustment of policy orientation, or unpredictable social and political dynamics. The intertwined influence of these complex factors may well be beyond the scope captured by the current model.

## Conclusion

This study reveals the key trends in the disease burden of uterine fibroids and endometriosis among reproductive-age women from 1990 to 2021 globally and in China, India, and the United States. The global disease burden of uterine fibroids continues to rise (mainly driven by population growth), while endometriosis shows a declining trend due to improvements in medical standards and awareness. The combined number of cases in China, India, and the United States accounts for nearly half of the global burden, and the distribution of the disease is significantly related to the level of SDI. The study found that, compared to developing countries (China, India), the peak age of uterine fibroid incidence in the United States is earlier, reflecting differences in dietary structure, reproductive patterns, and medical accessibility. It is necessary to pay attention to the peak age of incidence in different countries and develop targeted prevention and intervention measures. Although the global burden of endometriosis has generally decreased, the United States may face a resurgence of cases due to issues such as delayed childbirth, obesity, and genetics. Health inequalities persist, with high-SDI populations bearing a greater disease burden, but attention must also be paid to underdiagnosis in resource-poor areas. Predictions indicate that the burden of uterine fibroids will continue to grow, while the burden of endometriosis will generally decline (except in the United States). These findings emphasize the urgent need for targeted interventions, including early screening programs, equitable distribution of medical resources, and context-specific national strategies, to eliminate health disparities and mitigate the long-term impact of these diseases on global women’s health.

## Data Availability

The original contributions presented in the study are included in the article/supplementary material, further inquiries can be directed to the corresponding authors.

## Footnotes

1
http://ghdx.healthdata.org/
